# Impact of *AGT* rs5050(T>G) variants on associations between estradiol and angiotensinogen levels: Multi-Ethnic Study of Atherosclerosis (MESA)

**DOI:** 10.1371/journal.pone.0339786

**Published:** 2025-12-31

**Authors:** Karita C. F. Lidani, Patrick J. Trainor, Shubham Tomar, Erin D. Michos, Erin S. Morgan, Jerome I. Rotter, Xiuqing Guo, Sotirios Tsimikas, Andrew P. DeFilippis

**Affiliations:** 1 Division of Cardiovascular Medicine, Department of Medicine, Vanderbilt University Medical Center, Nashville, Tennessee, United States of America; 2 Division of Cardiology, Department of Medicine, Johns Hopkins University School of Medicine, Baltimore, Maryland, United States of America; 3 Ionis Pharmaceuticals, Carlsbad, California, United States of America; 4 The Institute for Translational Genomics and Population Sciences, Department of Pediatrics, The Lundquist Institute for Biomedical Innovation at Harbor-UCLA Medical Center, Torrance, California, United States of America; 5 Division of Cardiovascular Medicine, University of California-San Diego, La Jolla, California, United States of America; Guangdong Nephrotic Drug Engineering Technology Research Center, Institute of Consun Co. for Chinese Medicine in Kidney Diseases, CHINA

## Abstract

**Aims:**

Angiotensinogen plays an essential role in maintaining circulatory homeostasis. *AGT* rs5050(T > G) has been identified as a regulator of the transcription of *AGT* mRNA, with differential expression between sexes. We sought to determine if rs5050(T > G), an estrogen response element, modifies the relationship between estrogen and angiotensinogen levels.

**Methods:**

rs5050(T > G) was genotyped, and plasma angiotensinogen levels were measured in 4,831 MESA participants, including postmenopausal women, on hormone therapy (n = 709) or not (n = 1,551), and 2,581 men. Linear regression models were employed to determine the associations of angiotensinogen with rs5050(T > G) allele dosage; and to evaluate whether rs5050(T > G) modifies the association between estradiol and angiotensinogen, with a main effect term and interaction term between rs5050(T > G)*estradiol. Estimated marginal means (EMMs) were used to further evaluate the effect of estradiol on angiotensinogen across different rs5050 alleles (T > G).

**Results:**

rs5050*TT* had the highest median levels of angiotensinogen, followed by *TG* and *GG*. Adjusted main effect model showed positive associations between estradiol and angiotensinogen, with each rs5050*T* allele associated with 0.329 SD higher log-angiotensinogen levels (CI 95% 0.293, 0.365). The interaction rs5050(T > G)*estradiol was not significant, with EMMs exhibiting overlapping slope confidence intervals across genotypes. The proportion of the variance in angiotensinogen explained by modeling increases from 47.9% to 51.6% when including rs5050(T > G) or interation rs5050(T > G)*estradiol in the model.

**Conclusions:**

rs5050(T > G) is associated with circulating angiotensinogen levels, but rs5050(T > G) alleles do not influence the relationship between estradiol and angiotensinogen. This suggests that estrogen’s effect on angiotensinogen regulation occurs independently of rs5050(T > G), despite its location within an estrogen-responsive element.

## Introduction

Angiotensinogen plays a central role in the renin-angiotensin-aldosterone system (RAAS) in regulating blood volume and pressure homeostasis. Several studies have demonstrated a positive association between plasma angiotensinogen levels and blood pressure (BP) [1,2]. Estrogen has been recognized as a potential contributor to expression and activity of angiotensinogen through multiple mechanisms, including the presence of estrogen-responsive transcriptional factor binding to the angiotensinogen (*AGT*) gene promoter. Notably, transcriptional factors such as USF1 (upstream stimulatory factor 1), Arp-1 (actin-related protein), Sp1 (specificity protein 1), and ER1 (estrogen receptor 1) have been found to directly or indirectly respond to estrogen, affecting the expression of angiotensinogen [3,4].

Single nucleotide polymorphisms (SNPs) in the transcription factor binding region (−25 to −1) of the *AGT* gene promoter have been reported to affect its expression [5], potentially influencing the development of essential hypertension [2]. *In vitro* studies demonstrated that the variant rs5050 (T > G, frequently referred to in the literature as A-20C), a thymidine to guanosine substitution at nucleotide –20 of the 5´UTR of the human *AGT* gene modifies the binding of ER1 to the *AGT* promoter, inducing an increase in *AGT* transcription after estrogen treatment [6]. However, the association of this variant with gene transcription, and estradiol levels in humans remains controversial [5,7–9].

Although studies have examined the relationship between the rs5050(T > G) variant and angiotensinogen levels, the interaction between this SNP, located in an estrogen response element, and angiotensinogen is limited. Therefore, the objective of our study was to determine whether the *AGT* rs5050(T > G) variant modifies the relationship between estrogen and angiotensinogen levels.

## Materials and methods

### Study participants and design

**Fig 1:**
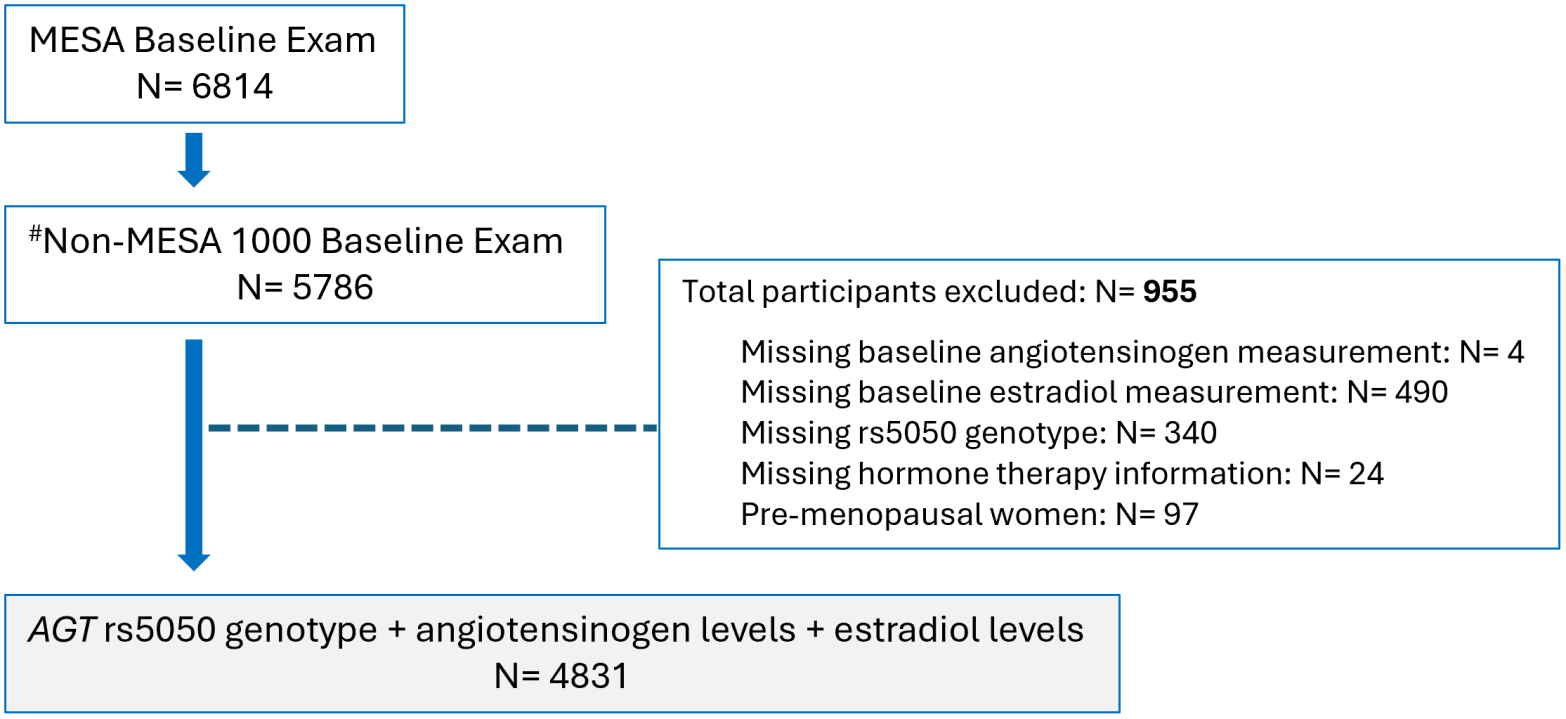
Participant inclusion/exclusion in study. Of 6,814 enrolled participants, 5,786 with measured angiotensinogen levels were eligible. After excluding 955 participants with missing key data (angiotensinogen, estradiol, rs5050 genotype, or hormone therapy) and premenopausal women, 4,831 participants remained for analysis. Note: ^#^ The MESA-1000 is a random sample of approximately 1,000 participants that has undergone additional tests using baseline blood samples. To minimize depletion of these specimens, ancillary studies such as this one typically exclude the MESA-1000 participants, referred to herein the Non-MESA 1000.

The Multi-Ethnic Study of Atherosclerosis (MESA) is an ongoing, population-based, longitudinal prospective study that enrolled 6,814 participants from July 2000 to July 2002. Participants, aged 45–85 years, were free of clinical cardiovascular disease (MI, angina, stroke, transient ischemic attack, heart failure, atrial fibrillation, revascularization, valve replacement, pacemaker or defibrillator implantation, or taking nitroglycerin) at enrollment. The design of the study has been described in detail in an earlier publication [10]. Our study population consisted of 4,831 individuals (2,250 postmenopausal women and 2,581 men) (**Fig 1).** The Institutional Review Boards of the participating sites approved the study, and all participants gave written informed consent. Additionally, MESA Publications and Presentations committee approved the conduct of the data analysis presented in this manuscript.

The MESA dataset was obtained from the MESA coordinating center (https://www.mesa-nhlbi.org/) at the University of Washington on 9/19/2024 and was analyzed at Vanderbilt University Medical Center. Availability of the MESA dataset is controlled by the MESA coordinating center. However, there is also a publicly available dataset that can be accessed. We would direct readers and reviewers to the publicly available datasets for MESA available from the NIH at: https://biolincc.nhlbi.nih.gov/studies/mesa/.

Women were considered postmenopausal if they were older than 55 years of age or had undergone a bilateral oophorectomy and/or self-reported being postmenopausal or experiencing absence of menstrual periods in the preceding year [11]. HT consisted of Premarin or Estratab (estrogen alone) (n = 232, 32.72%) or Premarin plus Provera, Estratab plus Provera, Prempro, or Premphase (estrogen combined with progesterone) (n = 91, 12.84%), while 54% (n = 386) had no data on the type of HT.

### Genotype data

The single nucleotide polymorphisms (SNPs) included in this study were obtained from 4,831 consenting MESA participants from the National Heart, Lung, and Blood Institute SNP Health Association Resource (SHARe) project. Non-typed SNPs were imputed using IMPUTE v2.2.2 to the 1000 genomes cosmopolitan Phase 1 v3 as a reference. Allele frequencies were calculated separately within each racial/ethnic group, and only those SNPs with minor allele frequencies (MAF) >0.01 were included in genetic association analyses. We further filtered imputed SNPs based on imputation quality >0.5, using the observed versus expected variance quality metric, and retained genotyped SNPs if their Hardy–Weinberg equilibrium p-value was ≥ 10^−5^.

### Angiotensinogen and estradiol levels

Circulating plasma angiotensinogen and serum estradiol (E2) levels were measured at the baseline (Exam 1) visit of the included MESA participants. Angiotensinogen measurements were made using an enzyme-linked immunoassay [12] and was executed by Medpace Reference Labs (Cincinnati, Ohio). The coefficient of variation was observed to be 9% with an analytical range of 13.9 µg/mL to 75.9 µg/mL. Total estradiol (E2) levels were measured from fasting serum samples at the University of Massachusetts Medical Center (Worcester, MA) by using an ultrasensitive radioimmunoassay kit (Diagnostic System Laboratories, Webster, TX) [11,13]. The intra-assay coefficient of variation was 10.5%.

### Statistical analysis

The distribution of cohort characteristics, including circulating angiotensinogen levels and estradiol levels, was determined within each rs5050(T > G) genotypes (GG, GT, TT). Angiotensinogen and estradiol levels were log-transformed given their right skewed distribution.

To assess the relationship between angiotensinogen and estradiol, we fit three linear regression models incorporating an additive genetic model. In this approach, rs5050(T > G) was coded as an allele dosage variable, representing the number of alleles (0, 1, or 2 copies of T allele). This coding allowed us to evaluate the impact of each additional *T* allele on the association between estradiol and angiotensinogen. In *Model A*, we regressed angiotensinogen levels on estradiol levels, adjusting for variables previously associated with angiotensinogen levels [2] that included age, sex by hormone therapy (HT) use (male, postmenopausal on HT, and postmenopausal not on HT), race/ethnicity, body mass index (BMI), total cholesterol, log-transformed high-sensitivity C-reactive protein (hs-CRP), log-transformed total testosterone, log-transformed dehydroepiandrosterone (DHEA), and log-transformed sex hormone-binding globulin (SHBG). Each analyte was scaled to have mean zero and standard deviation 1. *Model B* extends *Model A* to incorporate rs5050(T > G) allele dosage to identify any additive association between the rs5050(T > G) and angiotensinogen levels. In *Model C*, we included an interaction term [rs5050(T > G) allele dosage*estradiol] to Model B to assess if the effect of estradiol on angiotensinogen levels depending on the number of T alleles. We additionally calculated partial R² values for each predictor to quantify the proportion of unique variance in log-angiotensinogen explained by each variable after accounting for all others.

To further investigate allele dosage-specific differences in the estradiol-angiotensinogen relationship, we used estimated marginal means (EMMs) to analyze how angiotensinogen levels change with each unit increase in estradiol, considering the number of rs5050_*T* alleles present. Differences in these slopes across rs5050(T > G) variants were calculated to quantify the extent of effect modification.

### Secondary analysis

A sensitivity analysis was performed to assess the impact of supplemental estrogen on angiotensinogen levels excluding women on HT given previous evidence of their effect on angiotensinogen levels. This analysis aimed to determine the extent to which these medications influenced associations observed in the primary analysis.

The analyses reported in the current work were conducted using the R statistical language [14] (version 4.0.2) and the following packages: *emmeans, emtrends*, and *dplyr*.

## Results

Baseline demographics of the 4,831 participants stratified by rs5050(T > G) variants are reported in Table 1. Genotype frequencies of rs5050(T > G) are presented in S1 Table. Circulating levels of angiotensinogen differed significantly between rs5050(T > G) variants with rs5050*GG* genotype presenting lower angiotensinogen levels (median 16.1 µg/mL), followed by rs5050*GT* (median 18.5 µg/mL) and rs5050*TT* (median 20.4 µg/mL) (p < 0.001) (S2 Table)*.*

**Table 1:** Cohort characteristics of 4,831 MESA participants stratified by *AGT* rs5050(T > G) genotypes.

Characteristic	rs5050*TT* (N = 3,284)	rs5050*TG* (N = 1,378)	rs5050*GG* (N = 169)
**Mean age ± SD, years**	63.78 **± **9.95	63.97 ± 9.57	63.46 ± 9.82
**Sex by HT, n (%)**			
Male	1755 (53.44)	729 (52.90)	97 (57.40)
Postmenopausal not on HT	1058 (32.22)	436 (31.64)	47 (27.81)
Postmenopausal on HT	471 (14.34)	213 (15.46)	25 (14.79)
**Race/Ethnicity, n (%)**			
White	1268 (36.61)	524 (38.03)	73 (43.20)
Black	871 (26.52)	321 (23.29)	32 (18.93)
Chinese	440 (13.40)	175 (12.70)	14 (8.28)
Hispanic	705 (21.47)	358 (25.98)	50 (29.59)
**Diabetes, n (%)**	431 (13.14)	174 (12.63)	22 (13.02)
**Mean BMI ± SD, kg/m2**	28.13 ± 5.24	28.12 ± 5.18	28.86 ± 5.82
**Mean total cholesterol ± SD, mg/dL**	194.02 ± 35.31	195.41 ± 37.54	201.08 ± 35.59
**Mean HDL cholesterol ± SD, mg/dL**	50.69 ± 15.01	50.17 ± 14.55	48.96 ± 13.73
**Mean LDL cholesterol ± SD, mg/dL**	117.05 ± 31.14	118.26 ± 32.93	124.36 ± 33.32
**Mean systolic BP ± SD, mmHg**	127.61 ± 21.58	128.44 ± 21.68	125.46 ± 19.74
**Mean diastolic BP ± SD, mmHg**	72.03 ± 10.29	72.50 ± 10.29	72.53 ± 9.85
**Hypertension, n (%)**	1534 (46.71)	652 (47.31)	64 (37.87)
**Any hypertension medication, n (%)**	1269 (38.64)	539 (39.14)	64 (37.87)
**ACEi or ARB use, n (%)**	601 (18.30)	268 (19.45)	32 (18.93)
**Smoking history, n (%)**			
Never	1638 (50.03)	673 (49.09)	79 (46.75)
Former	1228 (37.51)	538 (39.24)	62 (36.69)
Current	408 (12.46)	160 (11.67)	28 (16.57)
**Alcohol history, n (%)**			
Never	695 (21.31)	267 (19.57)	25 (14.79)
Former	779 (23.89)	347 (25.44)	43 (25.44)
Current	1787 (54.80)	750 (54.99)	101 (59.76)
**Median hs-CRP [Q1, Q3], mg/L**	1.81 [0.82, 4.04]	1.86 [0.83, 4.0]	1.91 [0.86, 4.30]
**Mean eGFR ± SD**	73.56 ± 15.93	73.51 ± 15.48	74.33 ± 16.64
**Median estradiol [Q1, Q3], nmol/L**	0.10 [0.07, 0.14]	0.10 [0.07, 0.14]	0.10 [0.07, 1.14]
**Median angiotensinogen [Q1, Q3], µg/mL**	20.4 [17.70, 24.30]	18.5 [15.50, 22.73]	16.1 [13.35, 19.88]

Note: data are shown as mean and standard deviation (SD), n (%), or median [Q1, Q3].

BMI, body mass index; BP, blood pressure; HDL, high-density-lipoprotein; hs-CRP, high-sensitivity C-reactive protein; LDL, low-density-lipoprotein; eGFR; Q1, first quartile (25th percentile); Q3, third quartile (75th percentile).

After adjusting for sex by HT, race/ethnicity, BMI, total cholesterol, hs-CRP, total testosterone, DHEA, and SHBG (Model A) one standard deviation (SD) higher log-estradiol level was associated with a 0.208 SD increase in log-angiotensinogen, corresponding to 1.9 ng/mL in absolute angiotensinogen units (0.208 x 9.150) (Table 2, S3 Table). This association remained consistent after adjusting for rs5050(T > G) allele dosage, with each additional T allele corresponding to a 0.329 SD increase in log-angiotensinogen, equivalent to 3.0 ng/mL (0.329 x 9.150) (Model B) (Table 3, S3 Table). However, the relationship between angiotensinogen and estradiol didn’t differ significantly between rs5050(T > G) allele dosage (interaction p = 0.635) (Model C; Table 4). To facilitate interpretation in biological units, S3 Table reports the raw standard deviations of angiotensinogen and all predictors, along with the approximate real-unit equivalents of a one-SD change.

**Table 2:** Adjusted linear model regressing log-transformed angiotensinogen on log-transformed estradiol levels adjusting for confounders previously associated with angiotensinogen levels (Model A).

Variable	Estimate (β)	(95% CI)	p-value	Partial R^2^
**(Intercept)**	0.379	(0.271, 0.487)	<0.0001	--
**log(estradiol)**	0.208	(0.182, 0.234)	<0.0001	0.045
**PM not on HT**	−0.268	(−0.317, −0.219)	<0.0001	0.001
**PM on HT**	0.651	(0.584, 0.718)	<0.0001	0.039
***Hispanic***	0.010	(−0.026, 0.045)	0.5966	0.008
***Black***	−0.084	(−0.132, −0.036)	0.0006	0.004
***Chinese***	−0.051	(−0.089, −0.014)	0.0074	0.007
**BMI**	−0.009	(−0.014, −0.005)	<0.0001	0.003
**Total cholesterol**	0.117	(0.097, 0.138)	<0.0001	0.024
**hs-CRP**	0.122	(0.100, 0.145)	<0.0001	0.021
**Testosterone**	−0.206	(−0.263, −0.148)	<0.0001	0.009
**DHEA**	−0.056	(−0.078, −0.033)	<0.0001	0.004
**SHBG**	0.056	(0.030, 0.082)	<0.0001	0.004

Adjusted R^2^ = 0.479

AIC = 11283.82

BIC = 11375.39

Note: Linear regression models adjusted by sex and hormone therapy (HT) status [men, women on HT, and women not on HT], body mass index (BMI), total cholesterol levels, high-sensitivity C-reactive protein (hs_CRP), total testosterone, estradiol, dehydroepiandrosterone (DHEA), and sex hormone-binding globulin (SHBG). Each analyte was log-transformed and scaled to have mean zero and standard deviation 1. Partial R² represents the proportion of variance in the outcome uniquely explained by each predictor after adjusting for all other variables.

**Table 3:** Adjusted linear model regressing log-transformed angiotensinogen on log-transformed estradiol levels adjusting for confounders previously associated with angiotensinogen levels including rs5050(T > G) genotypes (Model B).

Variable	Estimate (β)	(95% CI)	p-value	Partial R^2^
**(Intercept)**	−0.181	(−0.304, −0.057)	0.0041	--
**log(estradiol)**	0.207	(0.181, 0.234)	<0.0001	0.048
**PM not on HT**	−0.268	(−0.317, −0.220)	<0.0001	0.001
**PM on HT**	0.685	(0.617, 0.752)	<0.0001	0.047
***Hispanic***	0.007	(−0.029, 0.043)	0.7071	0.007
***Black***	−0.097	(−0.144, −0.049)	<0.0001	0.004
***Chinese***	−0.031	(−0.068, 0.006)	0.0973	0.008
**BMI**	−0.008	(−0.013, −0.004)	0.0004	0.003
**Total cholesterol**	0.123	(0.102, 0.143)	<0.0001	0.028
**hs-CRP**	0.120	(0.098, 0.143)	<0.0001	0.022
**Testosterone**	−0.186	(−0.244, −0.128)	<0.0001	0.008
**DHEA**	−0.055	(−0.078, −0.032)	<0.0001	0.005
**SHBG**	0.050	(0.024, 0.076)	0.0002	0.003
**rs5050(*T > G)***	0.329	(0.293, 0.365)	<0.0001	0.062

Adjusted R^2^ = 0.516

AIC 10216.28

BIC 10313.37

Note: Linear regression models adjusted by sex and hormone therapy (HT) status [men, women on HT, and women not on HT], body mass index (BMI), total cholesterol levels, high-sensitivity C-reactive protein (hs_CRP), total testosterone, estradiol, dehydroepiandrosterone (DHEA), sex hormone-binding globulin (SHBG), and rs5050(T > G). Each analyte was log-transformed and scaled to have mean zero and standard deviation 1. Partial R² represents the proportion of variance in the outcome uniquely explained by each predictor after adjusting for all other variables.

**Table 4:** Adjusted linear model regressing log-transformed angiotensinogen on log-transformed estradiol levels adjusting for confounders previously associated with angiotensinogen levels including rs5050(T > G) genotypes and an interaction term between rs5050(T > G) and log(estradiol) (Model C).

Variable	Estimate (β)	(95% CI)	p-value	Partial R^2^
**(Intercept)**	−0.180	(−0.304, −0.057)	< 0.0001	---
**log(estradiol)**	0.208	(0.181, 0.234)	0.021	0.009
**PM not on HT**	−0.268	(−0.317, −0.219)	< 0.0001	0.001
**PM on HT**	0.685	(0.618, 0.752)	0.012	0.047
***Hispanic***	0.007	(−0.029, 0.043)	0.005	0.006
***Black***	−0.096	(−0.144, −0.049)	0.192	0.004
***Chinese***	−0.031	(−0.068, 0.006)	< 0.0001	0.008
**BMI**	−0.008	(−0.013, −0.004)	0.0005	0.003
**Total cholesterol**	0.123	(0.102, 0.143)	< 0.0001	0.028
**hs-CRP**	0.120	(0.098, 0.143)	< 0.0001	0.022
**Testosterone**	−0.186	(−0.244, −0.128)	< 0.0001	0.008
**DHEA**	−0.055	(−0.078, −0.032)	< 0.0001	0.005
**SHBG**	0.050	(0.024, 0.076)	0.0002	0.003
**rs5050(*T > G)***	0.329	(0.293, 0.366)	< 0.0001	0.062
rs5050(T > G)*log(estradiol)	−0.009	(−0.046, 0.028)	0.635	<0.001

Adjusted R^2^ = 0.516

AIC = 10218.07

BIC = 10321.62

Note: Linear regression models adjusted by sex and hormone therapy (HT) status [men, women on HT, and women not on HT], body mass index (BMI), total cholesterol levels, high-sensitivity C-reactive protein (hs_CRP), total testosterone, estradiol, dehydroepiandrosterone (DHEA), sex hormone-binding globulin (SHBG), rs5050(T > G), and interaction term between rs5050(T > G) and log(estradiol). Each analyte was log-transformed and scaled to have mean zero and standard deviation 1. Partial R² represents the proportion of variance in the outcome uniquely explained by each predictor after adjusting for all other variables.

**Fig 2:**
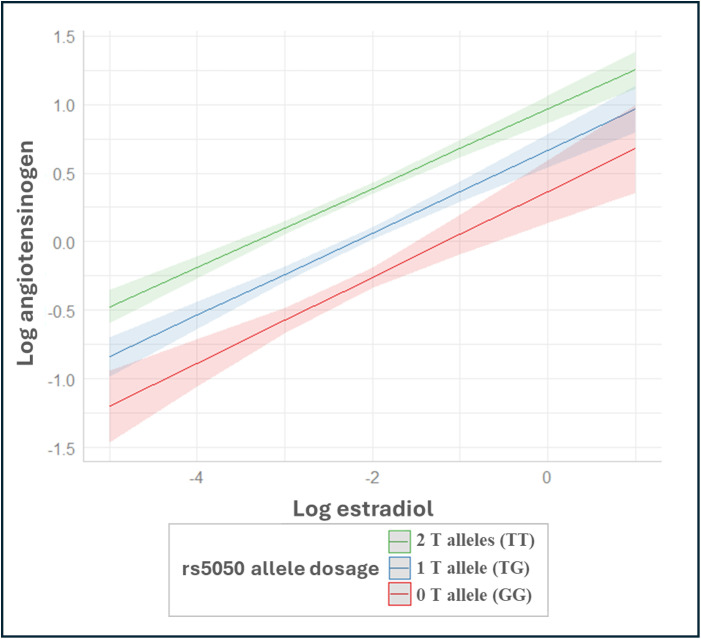
Relationship between log-transformed estradiol and log-transformed angiotensinogen levels in 4,831 MESA participants (2,250 postmenopausal women and 2,581 men), based on linear regression analysis using estimated marginal means (EMM). Note: Estimated marginal means from linear regression analysis of angiotensinogen levels on estradiol levels stratified by rs5050(T > G), adjusted for rs5050(T > G) allele dosage (TT = 2 T alleles, TG = 1 T allele, and GG = no T allele), interaction between rs5050(T > G) allele dosage*estradiol, age, sex by hormone therapy (HT) use (male, postmenopausal on HT, and postmenopausal not on HT), race/ethnicity, body mass index (BMI), total cholesterol, log-transformed high-sensitivity C-reactive protein (hs-CRP), log-transformed total testosterone, log-transformed dehydroepiandrosterone (DHEA), and log-transformed sex hormone-binding globulin (SHBG).

Fig 2 illustrates the positive association between estrogen and angiotensinogen, stratified by rs5050(T > G) allele T dosage (Model C). While levels of angiotensinogen vary by rs5050(T > G) allele dosage, the EMM analysis showed no evidence that the relationship between estrogen and angiotensinogen differed across rs5050(T > G) [slope 0.222 (95% CI: 0.155, 0.289) for GG, 0.213 (95% CI: 0.178, 0.249) for GT, and 0.204 (95% CI: 0.175, 0.233) for TT].

The proportion of the variance in angiotensinogen explained by the adjusted Model A which includes the variables noted above but excludes rs5050(T > G) variants is 47.9%. This proportion increases to 51.6% when including the rs5050(T > G) (Model B); and remains at 51.6% with the addition of the interaction term rs5050(T > G)*estradiol (Model C) (Tables 2–4).

Sensitivity analysis excluding postmenopausal women on HT showed lower association coefficient for estradiol compared to results in the primary analysis for all models (S4 Table).

## Discussion

This study contributes and expands upon the existing literature on angiotensinogen, estradiol, and rs5050(T > G) as follows: (1) median angiotensinogen levels were highest in rs5050 *TT* genotype compared to rs5050 *TG* and rs5050 *GG*; (2) no evidence of significant interaction between rs5050(T > G)*estradiol or genotype-specific differences in angiotensinogen-estradiol slopes was observed; (3) the proportion of the variance in circulating angiotensinogen levels increased slightly from 47.9% to 51.6% with the inclusion of rs5050(T > G) genotype alone or along with the interaction term rs5050(T > G)*estradiol. These findings highlight the complex genetic and hormonal regulation of angiotensinogen, with implications for understanding blood pressure regulation and related cardiovascular risks.

Although both rs5050(T > G) variants and estradiol levels were independently associated with circulating angiotensinogen, the regulation of *AGT* gene expression by rs5050 (T > G) variants does not appear to be influenced by estrogen levels. While estradiol is positively associated with angiotensinogen levels across all rs5050(T > G) genotypes, the presence of estrogen response elements in the *AGT* promoter region [15,16] suggests that estrogen may play a role in angiotensinogen expression. However, the transcription factor Arp-1 also binds to the rs5050(T > G) and reduces estrogen-induced promoter activity by competitively inhibiting ER binding and antagonizing the effects of the estrogen receptor-alpha [3,17]. Therefore, estrogen-induced expression of *AGT* gene (containing allele *T*) may depend on the amount of functional Arp-1 and ER present in different cells. Moreover, recent reports have provided evidence that the relationship between rs5050(T > G) polymorphism and angiotensinogen levels is via the binding of two transcription factors, upstream stimulatory factor 1 and 2, to the E box sequence that the SNP is in [3,18], rather than via estrogen receptor binding.

Conversely, substantial evidence indicates that sex hormones, particularly estrogen, significantly influence angiotensinogen expression. Some studies suggest that the association between *AGT* variants and circulating angiotensinogen levels may be sex-specific, with estrogen playing a key role in *AGT* regulation [2,19]. *In vitro* studies have demonstrated that the rs5050(T > G) variant alters the binding affinity of estrogen receptor 1 (ER1) to the *AGT* promoter, leading to increased AGT transcription in human liver cells following estrogen treatment [6]. Schunkert et al. [15] described that the effect of HT on increased angiotensinogen is involved in the regulation of this protein or its gene expression. This may be explained by the fact of *AGT* promoter gene is controlled in part by estrogen through estrogen response elements [20].

There are some limitations to this study. We restricted our sample to post-menopausal women as sex hormone levels differ between pre- and postmenopausal women, and there were relatively few pre-menopausal women in MESA (17%), which would limit analyses in this subset. Route and dosage of HT were not available for this analysis, both of which may impact on angiotensinogen levels. Lastly, while we adjusted for known confounders, there may be variable effects of environmental factors, such as lifestyle or additional hormonal influences, that could affect the observed associations.

## Conclusion

The rs5050(T > G) is significantly associated with angiotensinogen levels, however, rs5050_T allele dosage does not modify the relationship between estrogen and angiotensinogen.

## Supporting information

S1 Table *AGT* rs5050(T > G) genotypes and alleles frequencies [n (%)] in males, postmenopausal (PM) not on hormone therapy (HT); and postmenopausal on HT. Note: The population is in Hardy-Weinberg equilibrium.
(DOCX)

S2 Table *AGT* rs5050(T > G) genotypes and circulating levels of estradiol and angiotensinogen by sex/HT. Note: data are shown as median [Q1, Q3]. AGT, angiotensinogen levels; PM, postmenopausal women; HT, hormone therapy.
(DOCX)

S3 Table Standard deviations of study variables in raw and log-transformed units. Means and standard deviations (SD) are shown for each variable in their original units and after natural log transformation. These SDs provide a reference for interpreting standardized regression coefficients, which represent the expected change in SDs of log-angiotensinogen per 1 SD increase in the predictor. Note: 1 SD values are reported in raw units and log-transformed units. Standardized regression coefficients reflect the change in SDs of log-angiotensinogen per 1 SD increase in each predictor.
(DOCX)

S4 Table Sensitivity analysis- Linear models regressing log-transformed angiotensinogen on log-transformed estradiol excluding postmenopausal women on hormone therapy (HT). (DOCX)
