# Supplementary material for: Impact of AGT rs5050(T>G) variants on associations between estradiol and angiotensinogen levels: Multi-Ethnic Study of Atherosclerosis (MESA)
Source: PLoS One. 2025 Dec 31;20(12):e0339786. doi: 10.1371/journal.pone.0339786 (PMC12755761; doi:10.1371/journal.pone.0339786)
Supplement: S1 Table — Note: The population is in Hardy-Weinberg equilibrium. (DOCX) [file pone.0339786.s001.docx]

**S1 Table***.* ***AGT* rs5050(T>G) genotypes and alleles frequencies [n (%)] in males, postmenopausal not on hormone therapy (HT); and postmenopausal on HT.**

| ***AGT* rs5050(T>G)** | **Male**  **N= 2501** | **PM not on HT**  **N= 1541** | **PM on HT**  **N= 709** |
| --- | --- | --- | --- |
| *TT genotype*, n (%) | 1755 (68.0) | 1058 (68.7) | 471 (66.4) |
| *TG genotype*, n (%) | 729 (28.2) | 436 (28.3) | 213 (30.1) |
| *GG genotype*, n (%) | 97 (3.8) | 47 (3.0) | 25 (3.5) |
| ***Total*** | 2581 | 1541 | 709 |
| *T allele*, n (%) | 4239 (82.1) | 2442 (82.8) | 1155 (81.5) |
| *G allele*, n (%) | 923 (17.9) | 530 (17.2) | 263 (18.5) |

| Note: The population is in Hardy-Weinberg equilibrium |
| --- |
