# Supplementary material for: Impact of AGT rs5050(T>G) variants on associations between estradiol and angiotensinogen levels: Multi-Ethnic Study of Atherosclerosis (MESA)
Source: PLoS One. 2025 Dec 31;20(12):e0339786. doi: 10.1371/journal.pone.0339786 (PMC12755761; doi:10.1371/journal.pone.0339786)
Supplement: S2 Table — Note: data are shown as median [Q1, Q3]. AGT, angiotensinogen levels; PM, postmenopausal women; HT, hormone therapy. (DOCX) [file pone.0339786.s002.docx]

**S2 Table.** ***AGT* rs5050(T>G) genotypes and circulating levels of estradiol and angiotensinogen by sex/HT.**

| **rs5050** | **Male**  N=2,581 | | **PM not on HT**  N=1,541 | | **PM on HT**  N=709 | | **All participants**  N=4,831 | |
| --- | --- | --- | --- | --- | --- | --- | --- | --- |
|  | **AGT** | **Estradiol** | **AGT** | **Estradiol** | **AGT** | **Estradiol** | **AGT** | **Estradiol** |
| ***TT*** | 18.9  [16.8, 21.3] | 0.11  [0.09, 0.14] | 21.4  [18.7, 24.6] | 0.06  [0.04, 0.08] | 37.1  [26.6, 47.9] | 0.24  [0.12. 0.36] | 20.4  [17.7, 24.3] | 0.09  [0.07, 0.14] |
| ***TG*** | 16.5  [14.6, 19.4] | 0.11  [0.09, 0.14] | 19.7  [16.8, 22.9] | 0.06  [0.04, 0.08] | 34.3  [24.0, 44.7] | 0.22  [0.14, 0.37] | 18.5  [15.5, 22.8] | 0.09  [0.07, 0.14] |
| ***GG*** | 15.4  [12.8, 18.2] | 0.11  [0.09, 0.14] | 16.9  [23.7, 20.0] | 0.07  [0.04, 0.09] | 25.9  [14.7, 33.8] | 0.19  [0.06, 0.35] | 16.1  [17.6, 24.3] | 0.10  [0.07, 0.14] |

Note: data are shown as median [Q1, Q3]. AGT, angiotensinogen levels; PM, postmenopausal women; HT, hormone therapy.
