# Supplementary material for: Impact of AGT rs5050(T>G) variants on associations between estradiol and angiotensinogen levels: Multi-Ethnic Study of Atherosclerosis (MESA)
Source: PLoS One. 2025 Dec 31;20(12):e0339786. doi: 10.1371/journal.pone.0339786 (PMC12755761; doi:10.1371/journal.pone.0339786)
Supplement: S3 Table — Means and standard deviations (SD) are shown for each variable in their original units and after natural log transformation. These SDs provide a reference for interpreting standardized regression coefficients, which represent the expected change in SDs of log-angiotensinogen per 1 SD increase in the predictor. Note: 1 SD values are reported in raw units and log-transformed units. Standardized regression coefficients reflect the change in SDs of log-angiotensinogen per 1 SD increase in each predictor. (DOCX) [file pone.0339786.s003.docx]

**S3 Table. Standard deviations of study variables in raw and log-transformed units.**

Means and standard deviations (SD) are shown for each variable in their original units and after natural log transformation. These SDs provide a reference for interpreting standardized regression coefficients, which represent the expected change in SDs of log-angiotensinogen per 1 SD increase in the predictor.

| **Variable** | **Means** | **1 SD (raw units)** | **1 SD (log units)** |
| --- | --- | --- | --- |
| Angiotensinogen | 22.16 | 9.150 | 0.330 |
| Estradiol | 0.125 | 0.119 | 0.700 |
| BMI | 28.19 | 5.250 | 0.180 |
| Total Cholesterol | 194.44 | 36.010 | 0.185 |
| hs-CRP | 3.61 | 5.620 | 1.138 |
| Total Testosterone | 8.35 | 7.980 | 1.502 |
| DHEA | 12.81 | 7.180 | 0.537 |
| SHBG | 59.88 | 43.70 | 0.557 |

Note: 1 SD values are reported in raw units and log-transformed units. Standardized regression coefficients reflect the change in SDs of log-angiotensinogen per 1 SD increase in each predictor. Body mass index (BMI), high-sensitivity C-reactive protein (hs_CRP), dehydroepiandrosterone (DHEA), and sex hormone-binding globulin (SHBG).
