# Supplementary material for: Impact of AGT rs5050(T>G) variants on associations between estradiol and angiotensinogen levels: Multi-Ethnic Study of Atherosclerosis (MESA)
Source: PLoS One. 2025 Dec 31;20(12):e0339786. doi: 10.1371/journal.pone.0339786 (PMC12755761; doi:10.1371/journal.pone.0339786)
Supplement: S4 Table — (DOCX) [file pone.0339786.s004.docx]

**S4** **Table.** Sensitivity analysis- Linear models regressing log-transformed angiotensinogen on log-transformed estradiol excluding postmenopausal women on hormone therapy (HT).

| **Model** | **Sensitivity Analysis**  Excluding postmenopausal on HT | | |
| --- | --- | --- | --- |
|  | **Log(estradiol) Estimate** | **(95% CI)** | **p-value** |
| ***Model A*** | Main effect = 0.065 | (0.036, 0.094) | < 0.0001 |
| ***Model B*** | Main effect = 0.065 | (0.037, 0.094) | < 0.0001 |
| ***Model C*** | Interaction rs5050(T>G)*Log(estradiol)= 0.024 | (-0.017, 0.065) | 0.251 |

Note: Model A regressing angiotensinogen on estradiol was adjusted for sex and hormone therapy (HT) status [men, women on HT, and women not on HT], body mass index (BMI), total cholesterol levels, high-sensitivity C-reactive protein (hs_CRP), total testosterone, dehydroepiandrosterone (DHEA), and sex hormone-binding globulin (SHBG). Model B extends Model A by incorporating rs5050(T>G). Model C extends Model B by incorporating the interaction between rs5050(T>G) and estradiol. Each analyte was log-transformed and scaled to have mean zero and standard deviation 1.
